# 

**Published:** 1915-03

**Authors:** 


### ANNOUNCEMENTS.

TENNESSEE STATE DENTAL ASSOCIATION. The 48th Annual Meeting of this Association will be held June 24th to 26th, 1915, in Sewanee. Programme and special information will appear in later issues of the dental journals.

C. OSBORN RHEA, *Secretary,* Nashville, Tenn.

---

KENTUCKY STATE DENTAL ASSOCIATION. Will hold its annual meeting June 8th to 10th, in Ashland. So far as can be announced at this time the plan is to have a clinical programme of the "progressive type." A more complete programme will be announced later. All reputable dentists of adjoining states are cordially invited.

CHARLES R. SHOCKLETTE, *Secretary,*
"The Atherton," Louisville, Ky.

---

ILLINOIS STATE DENTAL SOCIETY. Will hold its annual meeting May 11th to 14th, in Peoria.

H. L. WHIPPLE, *Secretary,* Quincy.

---

KANSAS STATE DENTAL ASSOCIATION, will hold its annual meeting in Topeka, April 27th to 29th. Dr. T. P. Hinman of Atlanta, Ga., will be the special guest of the Association.

A. L. BENTON, *Secretary,*
Garnett, Kan.

MISSOURI STATE DENTAL ASSOCIATION, will celebrate its fiftieth anniversary June 10th to 12th. Special program next month. S. C. A. Rubey, *Secretary*,

Kansas City, Mo.

---

EXAMINATION OF DENTISTS FOR THE UNITED STATES ARMY.—Examinations of graduate dentists, between the ages of 21 and 27 years for positions as acting dental surgeons, will be held Monday, April 12, 1915, at the Columbus Barracks, Columbus, Ohio, and at Fort Slocum, N. Y.; Jefferson Barracks, Mo.; Fort Logan, Colorado, and Fort McDowell, California, on the same date. The contract is for three years. The salary, $150.00 per month, with certain furnishings and privileges. Applicants should write to the Surgeon General, Washington, D. C. at least two weeks before examination date for permission to take the examination. Send all data with application.

---

AMERICAN INSTITUTE OF DENTAL TEACHERS. —At the last annual meeting of the American Institute of Dental Teachers held at Ann Arbor, Michigan, the following officers were elected for the ensuing year: President, H. M. Semans, Columbus, Ohio; Vice-President, S. W. Bowles, Washington, D. C.; Secretary-Treasurer, J. F. Biddle, 517 Arch Street, N. S., Pittsburgh, Pa.; Executive Board, A. W. Thornton, Montreal, Canada; R. W. Bunting, Ann Arbor, Michigan; A. D. Black, Chicago, Ill.

The next annual meeting will be held at Minneapolis, Minnesota, January 25th, 26th, and 27th, 1916.

---

MICHIGAN STATE DENTAL SOCIETY.—Will meet in Grand Rapids, April 15th to 17th. Hotel Pantlid. Excellent program is in preparation.

F. W. Howlett, *Secretary*.

RELIEF OF SOLDIERS DENTALLY INJURED IN EUROPEAN WAR.—At the meeting of the American Institute of Dental Teachers held at Ann Arbor, Michigan, on January 26th, it was decided to take steps that should result in the raising of a fund to be used through the Red Cross Society, in giving relief and aid to the soldiers in Europe who are suffering from oral and dental injuries. The president was instructed to appoint a committee to take charge of this matter. President F. W. Gethro, under this instruction, appointed the following Executive and General committees:

Executive Committee—Henry W. Morgan, E. A. Johnson, Ellison Hillyer, John F. Biddle, Secretary, C. R. E. Koch, Chairman.

General Committee—E. C. Kirk, Philadelphia; J. H. Kennerly, St. Louis; H. C. Miller, Portland, Ore.; D. M. Gallie, Chicago; John F. Biddle, Pittsburgh; E. T. Darby, Philadelphia; Alfred Owre, Minneapolis; B. Holly Smith, Baltimore; E. A. Johnson, Boston; Frank Holland, Atlanta; D. M. Cattell, Memphis; Frederick R. Henshaw, Indianapolis; S. W. Bowles, Washington; E. H. Smith, Boston; A. H. Hipple, Omaha; Ellison Hillyer, New York; Truman W. Brophy, Chicago; D. H. Squire, Buffalo; H. E. Friesell, Pittsburgh; Henry W. Morgan, Nashville; I. N. Broomell, Philadelphia; Wallace Wood, New Orleans; Frank T. Breene, Iowa City; H. L. Banzhaf, Milwaukee; J. G. Sharp, San Francisco; G. V. Black, Chicago; W. T. Chambers, Denver; H. M. Seamans, Columbus; J. D. Patterson, Kansas City; N. S. Hoff, Ann Arbor; C. N. Johnson, Chicago; H. L. Wheeler, New York; L. E. Ford; Los Angeles; C. R. E. Koch, Chicago; H. B. Tileston, Louisville.

The Executive committee is contemplating the issue of contribution certificate booklets. Each booklet will contain twenty (20) certificates or coupons certifying that the holder thereof has contributed 25 cents to this fund. This certificate will be neatly lithographed, something like national currency. It will be printed in lilac ink—the color of the dental profession—and bear upon its face the Red Geneva cross.

It is hoped that the dental schools, dental students and dental societies, as well as the profession at large, will become sufficiently interested in this propaganda to secure a large enough fund, through these small contributions, to secure real relief for the class of war sufferers for which it is designed. That it may aid in the establishment of several special hospitals or wards devoted to dental and oral surgery injuries, within the belligerent zone of Europe, is the ultimate purpose of this movement.

It is expected that these booklets will be ready for distribution on or before March 1st. Applications for them may be made to Dr. John F. Biddle, of Pittsburgh, Pa., Secretary of the Executive committee; or to Dr. C. R. E. Koch, 31 West Lake St., Chicago, Chairman of the committee, before March 1st. After that date all the members of the Executive committee and General committee will be in a position to supply them.

---

OFFICERS OHIO STATE DENTAL SOCIETY—E. C. Mills, Columbus, president; T. I. Way, Cincinnati, first vice-president; F. M. Casto, Cleveland, second vice-president; F. R. Chapman, Columbus, secretary, and S. B. Ruggles, Portsmouth, treasurer.

The directors are: J. R. Callahan, Cincinnati; C. I. Keely, Hamilton; J. B. Stewart, Cincinnati; H. F. Harvey, Cleveland and J. D. Gordon, Cincinnati. The delegates to the national congress to attend for two years, are: L. L. Barber, Toledo; H. C. Brown, Columbus; L. E. Custer, Dayton, and J. R. Callahan, Cincinnati; and for one year, W. A. Price, Cleveland; F. M. Casto, Cleveland; C. W. Mills, Chillicothe, and E. C. Mills, Columbus.

